# Mitochondrial *COI* and *cytb* Characterisation of K‐Lineage *Varroa destructor* Isolates From Kastamonu, Türkiye

**DOI:** 10.1002/vms3.71084

**Published:** 2026-07-15

**Authors:** Mübeccel Atelge, Nuri Ercan, Abdullah İnci, Alparslan Yıldırım

**Affiliations:** ^1^ Department of Parasitology Faculty of Veterinary Medicine, Kastamonu University Kastamonu Türkiye; ^2^ Department of Food Technology, Kaman Vocational School Kırşehir Ahi Evran University Kırşehir Türkiye; ^3^ Department of Parasitology, Faculty of Veterinary Medicine Erciyes University Kayseri Türkiye

**Keywords:** *COI*, *cytb*, haplotype, phylogeny, Türkiye, *Varroa destructor*

## Abstract

**Background:**

*Varroa destructor* is the most important ectoparasitic mite of the western honeybee (*Apis mellifera*) and remains a major threat to apiculture worldwide. Molecular characterisation of local mite populations is essential for understanding lineage composition and for establishing sequence‐based epidemiological baseline data.

**Objectives:**

This study aimed to determine the haplotypes and phylogenetic relationships of representative *V. destructor* isolates collected from Kastamonu Province in the Western Black Sea region of Türkiye using mitochondrial *COI* and *cytb* markers.

**Methods:**

A total of 100 adult female mites were collected from 10 locations, and one representative high‐quality isolate from each locality (*n* = 10 per locus) was selected for bidirectional sequencing. Partial mitochondrial *COI* and *cytb* regions were amplified by PCR and sequenced. Consensus sequences were compared with GenBank records using BLASTn, and separate comparative datasets were constructed for each marker. Multiple‐sequence alignments were generated with MAFFT, substitution models were selected with ModelFinder, and phylogenetic relationships were inferred using Maximum‐likelihood and Bayesian approaches.

**Results:**

All sequenced Kastamonu representatives were completely identical within each marker, indicating mitochondrial homogeneity among the analysed isolates. In both the *COI* and *cytb* datasets, the study isolates clustered within the invasive K (Korean) lineage of *V. destructor*. In the *COI* analysis, the Kastamonu sequence grouped clearly with K‐lineage references and separately from J‐lineage sequences. The *cytb* analysis supported the same lineage assignment and likewise revealed no local mitochondrial subdivision within the sequenced representative subset. Comparative diversity analyses showed that, although the broader global datasets retained regional variation, the Kastamonu isolates analysed in this study were represented by a single mitochondrial background at both loci.

**Conclusion:**

The representative *V. destructor* isolates analysed from Kastamonu were identical at both mitochondrial markers analysed and belonged exclusively to the invasive K‐lineage. These findings provide sequence‐based confirmation of the maternal lineage detected among the sequenced mites and establish a regional molecular baseline for future surveillance of *V. destructor* populations in Türkiye. However, these findings should be interpreted within the limits of the representative sequencing design and should not be considered a definitive estimate of population‐wide genetic diversity.

## Graphical abstract

This study provides the molecular characterisation and phylogenetic analysis of *Varroa destructor* collected in Kastamonu, Türkiye. Using mitochondrial *COI* and *cytb* markers, the research identifies an identical K‐lineage, offering critical insights into the genetic diversity and evolutionary relationships within the region's mite populations.

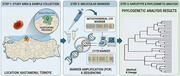

## 1 Introduction

Beekeeping, one of the oldest agricultural practices, is of major importance for both food security and ecosystem stability because the Western honeybee, *Apis mellifera*, is a key pollinator of wild and cultivated plants. In addition to their ecological role, honeybees provide economically valuable products such as honey, propolis, and royal jelly. However, *A. mellifera* populations have been declining worldwide under the combined pressure of habitat degradation, pesticide exposure, climate change, nutritional stress, and infectious and parasitic diseases. Among these stressors, parasitic infestations and pathogen transmission are among the most important causes of colony weakening and loss (Altay et al. 2025; Glenny et al. 2017; Lannutti et al. 2022; Utuk et al. 2022).

*Varroa destructor* (Acari: Varroidae) is regarded as the most damaging ectoparasite of honeybees and one of the principal biological threats to modern apiculture worldwide (Sammataro et al. 2000). Originally associated with the eastern honeybee, *Apis cerana*, this mite shifted to *A. mellifera* during the twentieth century and subsequently spread globally through apicultural trade and colony movements, causing severe biological and economic losses (Dietemann et al. 2019). The global invasion of *V. destructor* has largely been attributed to a limited number of successful maternal lineages, particularly those capable of reproducing efficiently on *A. mellifera*, which explains both its epidemiological success and the relatively low diversity reported in many invaded regions (Anderson and Trueman 2000; Navajas et al. 2010; Solignac et al. 2005). In Türkiye, the parasite was first reported in 1976 after entering through Bulgaria and then spread rapidly from Thrace to the rest of the country, largely through migratory beekeeping practices (Aydın et al. 2007; Balkaya et al. 2016).

Türkiye is one of the leading apicultural countries in the world and ranks among the top countries in terms of colony numbers (FAOSTAT 2023; TÜİK 2023). Nevertheless, as in many other parts of the world, *V. destructor* remains the most important parasitic threat to colony health and sustainable beekeeping in the country (Aydın et al. 2007; Mayack and Hakanoglu 2022; Seven Çakmak and Çakmak 2016; Sürsal Şimşek and Şimşek 2025). Because the spread, persistence, and control failure of this mite are closely linked to its population structure and dispersal history, sustainable management depends not only on physical, biological, and chemical control, but also on molecular epidemiological surveillance and genetic characterisation of circulating populations (Hua et al. 2023; Jeyapriya et al. 2025; Rosenkranz et al. 2010).

Understanding the genetic diversity of *V. destructor* is therefore essential for phylogenetic inference, epidemiological tracking, and the interpretation of regional spread patterns. Mitochondrial markers have played a central role in this field, and among them, *COI* and *cytb* are particularly informative for detecting haplotypic variation and reconstructing maternal lineages. Early studies demonstrated that the K and Japanese (J) haplotypes are the major lineages associated with infestation of *A. mellifera*, while subsequent analyses using broader mitochondrial datasets revealed additional Asian variants and highlighted the evolutionary significance of host‐shift‐related bottlenecks (Lannutti et al. 2022; Morfin et al. 2023; Navajas et al. 2010; Utzeri et al. 2019). More recent work has further shown that analysis based on concatenated or complementary *COI* and *cytb* data improves phylogenetic resolution, reveals cryptic variation, and helps identify region‐specific haplotypes and microevolutionary patterns that may remain undetected in single‐locus approaches (Gajić et al. 2013, 2016, 2019; Günyaktı Kılınç et al. 2025; Lin et al. 2021).

Previous studies from different parts of Türkiye have investigated the molecular identity of *V. destructor* populations using PCR‐RFLP and sequence‐based approaches (Altay et al. 2025; Ayan and Aldemir 2018; Ayan et al. 2017a, 2017b; Aydın et al. 2007; Gürler et al. 2024; Kesik et al. 2025; Koç et al. 2021; Utuk et al. 2022; Warrit et al. 2004). These investigations consistently support the predominance of the K haplotype in the country. In Kastamonu, likewise, only the K haplotype has been reported to date (Gürler et al. 2024; Warrit et al. 2004). However, despite this growing body of evidence, sequence‐based data from Kastamonu populations remain absent from GenBank, and multilocus mitochondrial characterisation of regional isolates is still lacking. This gap is important because even populations that appear homogeneous at first glance may contain informative polymorphisms or locally distributed variants detectable only through direct sequencing of more than one mitochondrial marker (Gajić et al. 2013, 2016; Günyaktı Kılınç et al. 2025; Lin et al. 2021).

Therefore, the present study aimed to characterise representative *V. destructor* isolates collected from different localities in Kastamonu Province by sequencing two mitochondrial markers, *COI* and *cytb*, in order to determine the mitochondrial sequence types detected among the analysed isolates, assess their phylogenetic relationships, and provide the first multilocus sequence records for this region to the GenBank database.

## 2 Materials and Methods

### 2.1 Study Area and Mite Collection

Kastamonu, located in the Western Black Sea region of Türkiye, is characterised by rugged topography and extensive forest cover. The province covers 13,108 km^2^ and has a mean elevation of approximately 775 m. Its rich forest structure, protected natural areas, and high plant diversity provide favourable conditions for apicultural activities.

**FIGURE 1:**
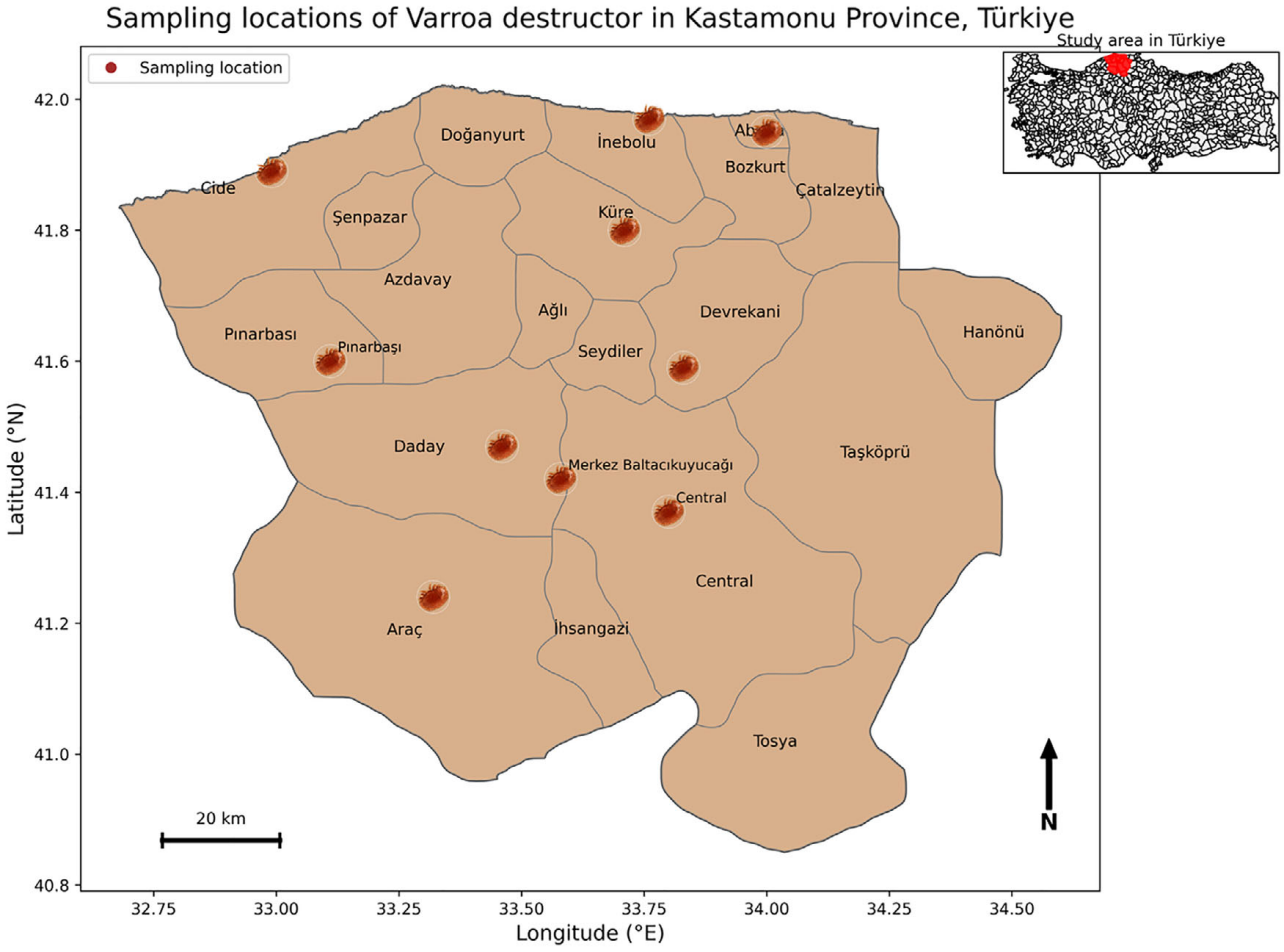
Map of the study area showing the collection sites of the *Varroa destructor* isolates.

A total of 100 adult female *V. destructor* mites were collected from 10 localities in Kastamonu province, with 10 mites sampled per locality, from apiaries where beekeeping is widely practiced (Figure 1). This sampling scheme was designed to obtain a geographically distributed mite collection across the study area and to ensure that each selected locality was represented in the subsequent molecular characterisation. All specimens were transported to the Parasitology Laboratory, Faculty of Veterinary Medicine, Kastamonu University, and preserved in 70% ethanol at −20°C until molecular analysis.

### 2.2 Genomic DNA Extraction

Before genomic DNA (gDNA) extraction, each mite was individually homogenised in a sterile microcentrifuge tube using a disposable pestle under liquid nitrogen. Genomic DNA was extracted using the PureLink Genomic DNA Mini Kit (Invitrogen, Thermo Fisher Scientific, USA) according to the manufacturer's instructions.

### 2.3 Amplification of the *COI* and *cytb* Regions

Two mitochondrial loci were targeted for molecular characterisation and phylogenetic analysis: a 376‐bp fragment of the *COI* gene amplified with the primer pair described by Solignac et al. (2005), and an approximately 985‐bp fragment of the *cytb* gene amplified with the primer pair described by Navajas et al. (2010). PCR was carried out in a final volume of 25 µL containing 2× DreamTaq Green PCR Master Mix (Thermo Scientific, K1081, USA), 0.5 µM of each primer, and 10–20 ng of template DNA. Thermal cycling conditions were identical for both loci and consisted of an initial denaturation at 95°C for 4 min, followed by 35 cycles of 95°C for 30 s, 52°C for 30 s, and 72°C for 1 min, with a final extension at 72°C for 10 min. PCR products were separated on 1.5% agarose gels and visualised using a CLP Gel Documentation System with GeneSnap software (Syngene, UVP Inc., Upland, CA, USA).

### 2.4 Sequencing and Sequence Editing

To represent the genetic profile of each sampling locality, one high‐quality amplicon per locality was selected for bidirectional Sanger sequencing for each marker (*n* = 10 per locus; Macrogen Europe). Although 100 adult female mites were collected to obtain a geographically distributed specimen set from Kastamonu Province, the sequencing component of the study was designed as a locality‐based representative molecular characterisation rather than a comprehensive population‐genetic survey. Therefore, considering the project scope, available sequencing budget, and the objective of generating regional baseline sequence data, one high‐quality representative mite from each locality was selected for bidirectional sequencing of each mitochondrial marker. Forward and reverse chromatograms were assembled and edited in Geneious Prime, and consensus sequences were generated after manual inspection of base calls and ambiguous positions. Sequence assembly and editing were performed following the general workflow implemented in Geneious Prime (Kearse et al. 2012).

### 2.5 Sequence Identification and Reference Retrieval

The resulting consensus sequences were queried against the NCBI GenBank nucleotide database using the BLASTn algorithm to confirm marker identity and to retrieve homologous reference sequences for comparative analyses (Altschul et al. 1990). For both markers, reference sequences were selected by considering BLAST similarity, locus correspondence, annotation quality, and overlap with the sequence region generated in this study.

### 2.6 Reference Sequence Selection and Dataset Curation

The *COI* and *cytb* datasets were analysed separately rather than as a concatenated matrix because locus‐specific reference selection, curation procedures, and rooting strategy differed between the two markers.

For the *COI* dataset, geographically diverse *V. destructor* reference sequences were selected from GenBank on the basis of BLAST similarity and manual curation. Only reference sequences overlapping the *COI* fragment generated in this study were retained in the curated comparative dataset. This approach was used to ensure that phylogenetic and diversity analyses were based on comparable *COI* sequence regions. The *Varroa jacobsoni* sequence AF106905, which overlapped with the study fragment, was included as the outgroup for the *COI* phylogenetic analyses. Because the *COI* fragment used in this study corresponds to the mitochondrial marker widely employed to distinguish invasive mitotypes of *V. destructor*, the curated *COI* dataset was used to assess the placement of the Kastamonu isolate within the K mitotype assemblage (Navajas et al. 2010; Solignac et al. 2005).

For the *cytb* dataset, the newly generated Kastamonu isolate PV033722 (KU‐VdesCytB) was used as the focal query sequence. Homologous *cytb* sequences were retrieved from GenBank using BLASTn, and approximately 20–25 geographically diverse *V. destructor* reference isolates were retained after manual inspection of sequence identity, query coverage, annotation quality, and locus correspondence. The *cytb* dataset was further curated by removing unsuitable or problematic records before diversity calculations. Because the most distant external record compressed ingroup branch‐length variation in preliminary phylogenetic visualisations, the final *cytb* trees were presented as midpoint‐rooted ingroup trees.

The regional diversity summaries were based on the curated comparative alignments used for phylogenetic analysis. These datasets included the newly generated representative Kastamonu sequence for each marker together with selected GenBank reference sequences; therefore, they should not be interpreted as diversity estimates for the 100 field‐collected mites or for the entire Kastamonu population. For *COI*, diversity indices were calculated using the comparable *V. destructor* dataset composed of sequences overlapping the study fragment, whereas the *V. jacobsoni* outgroup was used only for phylogenetic rooting and was not included in the diversity summary. For *cytb*, diversity indices were calculated using the corrected curated comparative dataset after removal of unsuitable/problematic records.

### 2.7 Multiple‐Sequence Alignment and Model Selection

Separate multiple‐sequence alignments were generated for *COI* and *cytb* using MAFFT v7 with the automatic strategy option (Katoh and Standley 2013). The best‐fitting nucleotide substitution model for each alignment was selected under the Bayesian Information Criterion (BIC) using ModelFinder as implemented in IQ‐TREE (Kalyaanamoorthy et al. 2017). Prior to diversity calculations, alignments were inspected manually and trimmed to a common comparable region to minimise the effects of unequal sequence lengths, missing data, and terminal gaps. For the *COI* dataset, the final comparative alignment used for diversity analysis was restricted to *V. destructor* sequences overlapping the *COI* fragment generated in this study. The *V. jacobsoni* outgroup used for *COI* phylogenetic rooting was not included in the *COI* diversity summary. For *cytb*, diversity calculations were performed using the corrected curated comparative alignment after removal of unsuitable or problematic records.

### 2.8 Phylogenetic Analyses

Phylogenetic analyses were performed independently for the *COI* and *cytb* datasets using both maximum‐likelihood (ML) and Bayesian inference (BI) approaches. ML trees were reconstructed in RAxML‐NG, and nodal support was assessed with 1000 bootstrap replicates (Kozlov et al. 2019). Bayesian analyses were conducted in MrBayes 3.2 using two independent runs with four Markov chains each under locus‐specific substitution settings inferred from model testing (Ronquist et al. 2012). For final tree visualisation, bootstrap support values ≥ 50 and posterior probabilities ≥ 0.50 were displayed. The *COI* trees were rooted using *V. jacobsoni* AF106905 as the outgroup. For the curated *cytb* ingroup dataset, midpoint rooting was used in the final figures to improve visualisation of among‐isolate relationships.

## 3 Results

Target amplicons of the mitochondrial *COI* and *cytb* loci were successfully obtained and bidirectionally sequenced from all examined *V. destructor* isolates. Sequence comparison among the 10 sequenced Kastamonu representatives showed complete identity within each marker, indicating that the analysed representative isolates represented a single mitochondrial sequence type for *COI* and likewise a single mitochondrial sequence type for *cytb*. Accordingly, one representative sequence per locus was used for comparative analyses and deposited in GenBank under accession numbers PV021105 (KU‐VdesCOI) and PV033722 (KU‐VdesCytB).

### 3.1 *COI* Sequence Diversity and Phylogenetic Relationships

**FIGURE 2:**
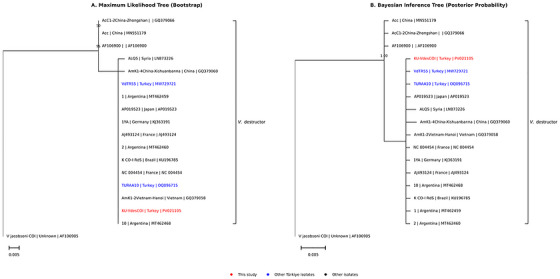
Maximum‐likelihood (ML) and Bayesian inference (BI) phylogenetic trees inferred from mitochondrial *COI* sequences of *V. destructor* isolates using the curated comparative alignment overlapping the *COI* fragment generated in this study. The newly generated Kastamonu isolate is shown in red, other Turkish isolates are shown in blue, and non‐Turkish reference isolates are shown in black. Bootstrap support values for the ML tree and posterior probabilities for the BI tree are indicated at the relevant nodes. The trees show the placement of the study isolate within the K mitotype of *Varroa destructor*. *Varroa jacobsoni* AF106905 was used as the outgroup.

When the Kastamonu *COI* sequence was analysed together with comparable GenBank references retained in the curated *COI* alignment, the Turkish study isolate was placed within the widely distributed *V. destructor* K mitotype assemblage (Figure 2). In both ML and BI analyses, the Turkish study isolate clustered with other Turkish sequences and with reference isolates from Europe, Asia, and South America within the larger *V. destructor* clade. The *V. jacobsoni* reference sequence AF106905 was used as the outgroup and was clearly separated from the *V. destructor* sequences after rooting. Overall, the ML and BI topologies were congruent and supported the assignment of the Kastamonu *COI* isolate to the K mitotype (Figure 2).

The regional diversity summary of the *COI* dataset is presented in Table 1. For the *COI* analyses, the curated comparative dataset was constructed using reference sequences that overlapped with the *COI* fragment generated in this study. Therefore, the diversity indices presented in Table 1 summarise variation within this comparable *COI* dataset and should not be interpreted as diversity estimates for all field‐collected mites or for the entire Kastamonu population. Therefore, the diversity indices presented in Table 1 summarise variation within the curated comparative *COI* dataset and should not be interpreted as diversity estimates for all field‐collected mites or for the entire Kastamonu population. Across all analysed *COI* sequences, 17 samples comprised 4 haplotypes, with 5 polymorphic sites, an average number of nucleotide differences (*k*) of 1.162, haplotype diversity (*hd*) of 0.493 (0.121), and nucleotide diversity (*Pi*) of 0.00374 (0.00450) (Table 1). The Turkish *COI* subset contained 3 sequences representing a single haplotype, with no segregating sites detected (*S* = 0), *k* = 0.000, *hd* = 0.000 (0.000), and *Pi* = 0.00000 (0.00000). In contrast, the Asian subset showed the highest variability, with 4 haplotypes among 5 sequences and 5 polymorphic sites (*k* = 2.000; *Pi* = 0.00643) (Table 1).

**TABLE 1:** Summary of molecular diversity indices of mitochondrial cytochrome c oxidase subunit I (*COI*) sequences.

Localities	*N*	No.	*S*	*k*	*hd* (±SD)	*Pi* (±SD)
Türkiye	3	1	0	0.000	0.000 (0.000)	0.00000 (0.00000)
Europe	3	1	0	0.000	0.000 (0.000)	0.00000 (0.00000)
Asia	5	4	5	2.000	0.900 (0.134)	0.00643 (0.00455)
South America	4	1	0	0.000	0.000 (0.000)	0.00000 (0.00000)
Other	2	1	0	0.000	0.000 (0.000)	0.00000 (0.00000)
**All samples**	17	4	5	1.162	0.493 (0.121)	0.00374 (0.00450)

### 3.2 *Cytb* Sequence Diversity and Phylogenetic Relationships

**FIGURE 3:**
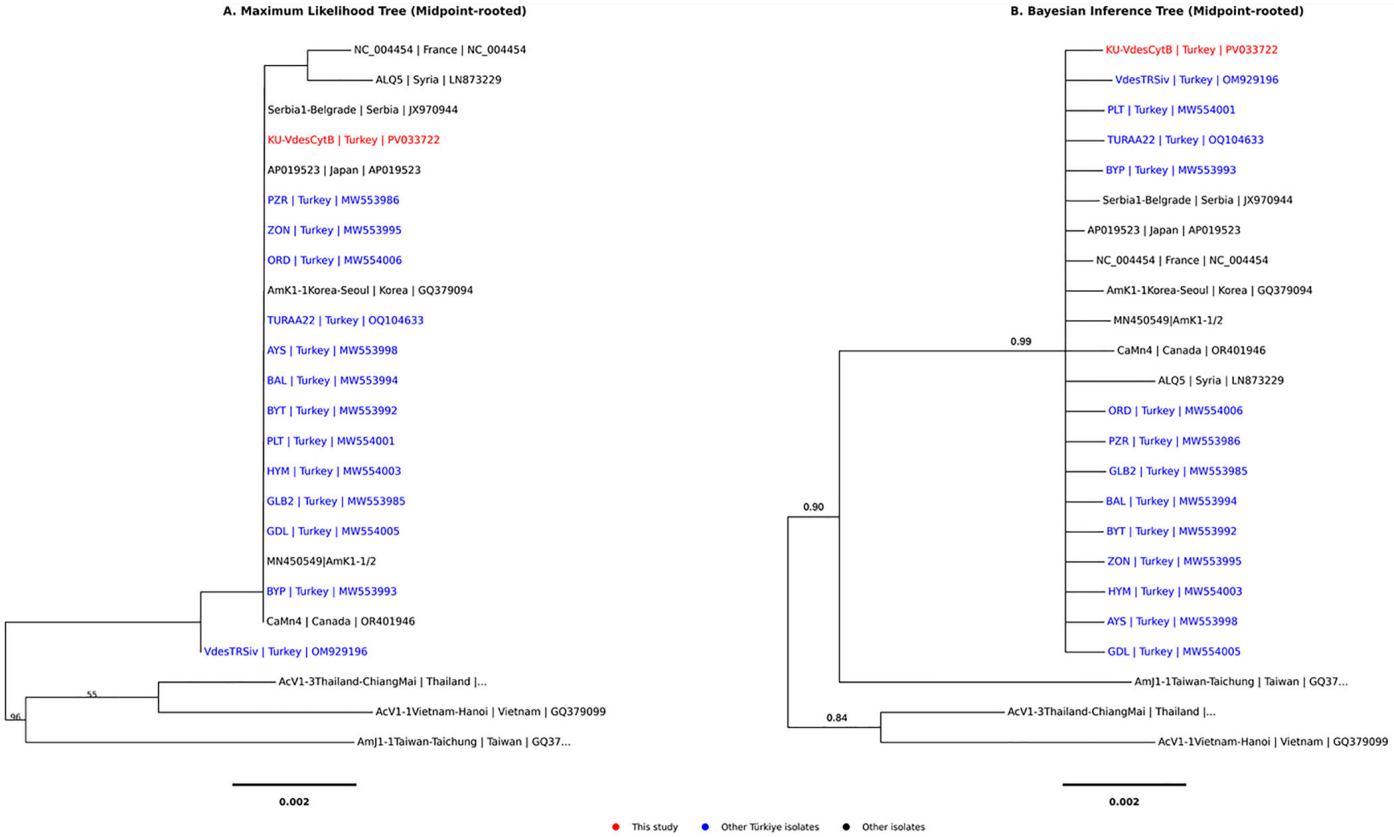
Maximum‐likelihood (ML) and Bayesian inference (BI) phylogenetic trees inferred from mitochondrial *cytb* sequences of *Varroa destructor* isolates using the curated comparative *cytb* alignment. The newly generated Kastamonu isolate is shown in red, other Turkish isolates are shown in blue, and non‐Turkish reference isolates are shown in black. Bootstrap support values for the ML tree and posterior probabilities for the BI tree are indicated at the relevant nodes.

The *cytb* dataset was analysed independently and, after curation of unsuitable reference records, the final ingroup tree was presented as a midpoint‐rooted phylogeny in order to improve visualisation of relationships among *V. destructor* isolates (Figure 3). As in the *COI* analysis, the Kastamonu *cytb* isolate grouped with other Turkish sequences and fell within the broader *V. destructor* assemblage rather than forming a distinct lineage. The ML and BI trees showed broadly concordant topologies, with the Turkish‐rich cluster placed together with isolates from Serbia, France, Syria, Japan, South Korea, and Canada, whereas several East and Southeast Asian references occupied more distant positions in the tree (Figure 3). Bayesian support for the principal internal groupings was moderate to high, with posterior probabilities of 0.99, 0.90, and 0.84 at the main internal nodes shown on the final tree, while the corresponding ML analysis recovered the same general branching pattern with comparable structure (Figure 3).

The regional diversity summary of the *cytb* dataset is shown in Table 2. As with the *COI* dataset, the *cytb* diversity indices were calculated from a curated comparative alignment containing the newly generated representative Kastamonu sequence and selected GenBank reference sequences. These values therefore summarise variation within the comparative dataset and should not be interpreted as estimates of genetic diversity among the 100 field‐collected mites or across the wider Kastamonu population. Across all analysed *cytb* sequences, 24 samples yielded 4 haplotypes and 8 polymorphic sites, with an average *k* value of 1.033, *hd* = 0.239 (0.087), and *Pi* = 0.00164 (0.00310) (Table 2). Within the Turkish *cytb* subset, 14 sequences represented a single haplotype, with no segregating sites detected (*S* = 0), *k* = 0.000, *hd* = 0.000 (0.000), and *Pi* = 0.00000 (0.00000). In contrast, the Asian subset showed the highest regional variability, with 4 haplotypes among 6 samples and 8 polymorphic sites (*k* = 3.400; *Pi* = 0.00541) (Table 2).

**TABLE 2:** Summary of molecular diversity indices of mitochondrial cytochrome b (*cytb*) sequences.

Localities	*N*	No.	*S*	*k*	*hd* (±SD)	*Pi* (±SD)
Türkiye	14	1	0	0.000	0.000 (0.000)	0.00000 (0.00000)
Europe	2	1	0	0.000	0.000 (0.000)	0.00000 (0.00000)
Asia	6	4	8	3.400	0.800 (0.163)	0.00541 (0.00339)
North America	1	1	0	NA	NA	NA
Other	1	1	0	NA	NA	NA
All samples	24	4	8	1.033	0.239 (0.087)	0.00164 (0.00310)

### 3.3 Overall Interpretation

Taken together, both mitochondrial markers support the placement of the sequenced representative Kastamonu isolates within the widespread K‐type *V. destructor* lineage. The *COI* analysis placed the Turkish study isolate within the K mitotype assemblage together with other Turkish and geographically diverse reference sequences, while *V. jacobsoni* AF106905 was clearly separated as the outgroup (Figure 2). The *cytb* analysis provided additional resolution among ingroup *V. destructor* sequences and showed that the Kastamonu isolate clustered with Turkish and western Eurasian reference sequences, whereas several East/Southeast Asian references occupied more distant positions in the tree (Figure 3). The diversity summaries indicate that the sequenced Kastamonu representatives were homogeneous for both mitochondrial markers, while the broader curated comparative datasets retained haplotypic and geographic variation (Tables 1 and 2).

## 4 Discussion

The present study showed mitochondrial uniformity among the sequenced representative Kastamonu *V. destructor* isolates at both *COI* and *cytb* loci. The representative sequences generated from different localities shared a single sequence type within each marker, and both phylogenetic datasets placed the study isolates within the widespread K‐type *V. destructor* lineage. This concordance across two mitochondrial markers strengthens the interpretation that the sequenced Kastamonu representatives belong to the globally invasive maternal background most commonly associated with *A. mellifera* infestations (Navajas et al. 2010; Lin et al. 2021).

Our findings are consistent with the long‐standing view that mitochondrial diversity in *V. destructor* populations established on *A. mellifera* is often limited by strong founder effects associated with host shift and subsequent spread. Navajas et al. (2010) showed that although substantial diversity exists in Asian populations overall, the mites reproducing on *A. mellifera* represent a narrower subset of that variation. In the same direction, the uniformity observed among the sequenced Kastamonu representatives is compatible with the bottleneck scenario proposed for the globally invasive lineages after transfer from *A. cerana* to *A. mellifera* (Navajas et al. 2010). However, given the representative sequencing design, these results should be interpreted as evidence of mitochondrial uniformity among the analysed isolates rather than as a definitive estimate of population‐wide diversity across Kastamonu.

The *COI* results are especially important because this marker placed the Kastamonu isolate within the K mitotype assemblage. This agrees with the broader literature showing that the K‐lineage remains the dominant invasive background in many *A. mellifera* populations worldwide (Gunyakti Kilinc et al. 2025; Navajas et al. 2010). At the same time, our results underline why direct sequencing remains essential. Mitochondrial uniformity alone does not identify the lineage; for example, Hua et al. (2023) found that all *A. mellifera*‐derived mites sampled in Taiwan were also identical at *COI*, but belonged to the J type rather than the K/R type. Thus, in our case, the conclusion depends not on homogeneity itself but on the phylogenetic placement of the Turkish sequence within the K‐type assemblage.

The *cytb* data support the same interpretation and add confidence to the conclusion. In our dataset, inclusion of a second mitochondrial marker did not reveal additional mitochondrial variation among the sequenced Kastamonu representatives; instead, it confirmed the same homogeneous pattern seen in *COI*. This is relevant because *cytb* has proven informative in studies where additional regional variants are present. For instance, Gajić et al. (2013) distinguished the Serbian S1 and P1 haplotypes from the K haplotype using single nucleotide differences in *COI* and *cytb*, and Gajić et al. (2016) later showed that these marker‐defined haplotypes could also coexist with mitochondrial heteroplasmy. The absence of comparable *cytb*‐defined variation among the analysed Kastamonu representatives suggests a simpler mitochondrial pattern in the sequenced material, at least at the mitochondrial level, than the more heterogeneous Serbian populations described to date.

This contrast with Serbia is particularly informative. In Serbia, sequencing‐based studies detected coexistence of the K haplotype with additional mitochondrial variants, and later work documented coexistence of multiple haplotypes within localities and even within colonies (Gajić et al. 2013, 2016, 2019). In our study, by contrast, neither *COI* nor *cytb* revealed evidence of coexisting maternal lineages among the sequenced representative isolates. This difference suggests that the Kastamonu isolates analysed here do not show the same mitochondrial heterogeneity reported from some southeastern European settings. Rather, they appear to represent a uniform K‐lineage mitochondrial background within the limits of the analysed material. In that respect, our findings provide a useful regional reference point for future Turkish surveillance studies that may test whether this pattern is maintained over time or differs among provinces.

The comparative diversity summaries also support this interpretation. In our study, the Turkish subsets were notably less variable than the broader global comparison sets, especially for *cytb*, whereas the overall datasets retained geographic diversity. This agrees with the broader in silico synthesis of Gunyakti Kilinc et al. (2025), who found considerable global haplotype diversity and regional structure when large GenBank‐derived *COI* and *cytb* datasets were analysed. Our results therefore should not be read as evidence that *V. destructor* is globally uniform, but rather that the sequenced Kastamonu representatives correspond to a locally uniform fraction of a broader and geographically structured species complex.

Another important point is methodological. In our material, *COI* and *cytb* converged on the same biological conclusion, but this does not reduce the value of using both markers. On the contrary, the agreement between them increases confidence that the observed uniformity among the sequenced representatives is not simply a consequence of limited resolution of a single locus. This is especially relevant in light of studies showing that additional mitochondrial structure can emerge when more than one marker is examined (Gajić et al. 2013; Navajas et al. 2010). Therefore, although our final result is one of mitochondrial uniformity among the analysed isolates, the multilocus approach remains justified because it allows a more robust assessment of marker‐specific variation.

From an epidemiological perspective, the detection of the K‐lineage in Kastamonu is important because this lineage has been strongly associated with the global expansion of *V. destructor* on *A. mellifera* (Navajas et al. 2010; Lin et al. 2021). Our study did not evaluate virulence or acaricide resistance directly, so those traits should not be inferred from mitochondrial identity alone. Nevertheless, documenting the presence of the K‐lineage in this region is still valuable because it establishes a sequence‐based baseline for future studies on population turnover, emergence of local variants, or possible introduction of additional lineages. In that sense, the main contribution of the present study is not the discovery of a novel haplotype, but the demonstration that the sequenced representative isolates from Kastamonu belong to the globally successful invasive K background and showed no detectable mitochondrial subdivision at the two loci examined.

## 5 Conclusion

In conclusion, the sequenced representative *V. destructor* isolates from Kastamonu showed mitochondrial uniformity at both *COI* and *cytb* loci and were assigned to the widely distributed invasive K lineage. No mitochondrial variation was detected among the analysed representatives at either marker. The congruent *COI* and *cytb* results indicate that the analysed Kastamonu isolates share a common K‐lineage maternal background, while the broader curated comparative datasets retain haplotypic and geographic diversity.

Although no novel haplotype was identified, this study provides validated *COI* and *cytb* sequence data from a region where direct nucleotide‐based evidence has been limited. These data establish a regional molecular baseline for future surveillance studies and may support monitoring of temporal shifts, emergence of local variants, or introduction of additional mitochondrial backgrounds into Turkish *V. destructor* populations.

## Author Contributions

Conceptualisation: Mübeccel Atelge, Alparslan Yıldırım. Methodology: Mübeccel Atelge, Nuri Ercan, Abdullah İnci, Alparslan Yıldırım. Investigation: Mübeccel Atelge, Nuri Ercan. Data curation: Mübeccel Atelge, Nuri Ercan. Formal analysis: Mübeccel Atelge, Nuri Ercan, Alparslan Yıldırım. Supervision: Mübeccel Atelge, Alparslan Yıldırım. Visualisation: Mübeccel Atelge, Nuri Ercan, Alparslan Yıldırım. Writing – original draft: Mübeccel Atelge. Writing – review & editing: Mübeccel Atelge, Nuri Ercan, Abdullah İnci, Alparslan Yıldırım.

## Funding

This research has been supported by Kastamonu University Scientific Research Projects Coordination Unit. Project Number: KÜBAP‐01/2023‐26.

## Ethics Statement

The authors confirm that the ethical policies of the journal, as noted on the journal's author guidelines page, have been adhered to. No ethical approval was required because the study involved the collection and molecular characterisation of ectoparasitic mites and did not include experimental procedures on vertebrate animals.

## Conflicts of Interest

The authors declare no conflict of interest.

## Data Availability

The sequence data generated in this study have been deposited in GenBank under accession numbers PV021105 (KU‐VdesCOI) and PV033722 (KU‐VdesCytB). Additional data supporting the findings of this study are available from the corresponding author upon reasonable request.
